# CAMPEP graduate program standards should require a dedicated course in Magnetic Resonance Imaging physics

**DOI:** 10.1002/acm2.12332

**Published:** 2018-04-25

**Authors:** David W. Jordan, Jing Cai, Yi Rong

**Affiliations:** ^1^ Department of Radiology University Hospitals Cleveland Medical Center Cleveland OH 44106 USA; ^2^ Department of Radiology Case Western Reserve University Cleveland OH 44106 USA; ^3^ Department of Health Technology and Informatics Hong Kong Polytechnic University Kowloon Hong Kong; ^4^ Department of Radiation Oncology University of California Davis Comprehensive Cancer Center Sacramento CA 95817 USA

## 1 INTRODUCTION

In a previous parallel opposed editorial, a heated debate was conducted on the topic whether an in‐house diagnostic Magnetic Resonance Imaging (MRI) physicist is needed for a radiation oncology department, considering the increased use of MRI‐guided radiotherapy.^1^ Interestingly, upon being presented with this topic, the therapy physicist chose to argue for the proposition, while the MRI physicist argued against. As a therapeutic physicist myself, I attribute that to the human nature of “we all fear what we do not understand.” The use of MRI infiltrates every aspect of radiotherapy, that is, diagnosis and staging, target definition, treatment planning, and more recently on‐board image guidance for treatment delivery. The more we employ MRI in radiotherapy, the more we feel short in our fund of knowledge of MRI physics. Undoubtedly, “hiring an in‐house diagnostic MR physicist” would alleviate this feeling of inadequacy, but it is logistically difficult to justify from a practical standpoint, considering the substantial financial commitment of hiring a dedicated MRI physicist to complement our staff of therapy physicists. As pointed out by the opposing side, one viable alternative would be to join in collaboration with colleagues in radiology when MRI expertise is needed.^1^ Yet that also involves logistical considerations in terms of shared cost for the personnel, responsibility, liability, scheduling, etc. Considering most therapeutic physicists lack a full and satisfactory MR knowledge, would it be an appropriate response for CAMPEP to mandate dedicated MRI courses within graduate medical physics programs? Herein, Dr. David Jordan is in support of the idea that “CAMPEP graduate program standards should require a dedicated course in Magnetic Resonance Imaging physics,” while Dr. Jing Cai argues against it.

Dr. David Jordan is a senior medical physicist at University Hospitals Cleveland Medical Center and Associate Professor of Radiology at Case Western Reserve University in Cleveland, Ohio. He is certified in Diagnostic Medical Physics, Nuclear Medicine Physics, and MRI Physics, and as a MR Safety Expert. He is active as the chair of the MR subcommittee for AAPM and serves as a board member for the American Board of Magnetic Resonance Safety and the International Accreditation Commission (IAC) MRI Accreditation Program. His main interests are MRI, dual‐energy CT, nuclear medicine, and radiology and medical physics resident education.

Dr. Jing Cai is currently an Associate Professor at Hong Kong Polytechnic University. Dr. Cai received his PhD degree in Engineering Physics in 2006 and completed his Medical Physics residency in 2009 from the University of Virginia. Afterward, Dr. Cai joined Duke University from 2009 to 2017 as a faculty physicist. Dr. Cai has published more than 70 referred journal articles and over 200 conference abstracts. Dr. Cai's research is focused on developing and clinically implementing novel image‐guided radiation therapy (IGRT) techniques, with an emphasis on MRI, and has received a number of federal, charitable, and industrial funding.

## 2 OPENING STATEMENTS

### 2.A David W. Jordan, PhD

MRI is an area of increasing importance for all clinical medical physicists, but at present, there is wide variation among CAMPEP graduate programs’ requirements for, or even offerings of, dedicated MRI coursework to prepare graduates for residency and practice. For therapeutic medical physicists, recent AAPM meeting programs reveal the rapid growth of interest in MRI education in support of MR‐assisted and/or MR‐based treatment planning, assessment, and monitoring applications as well as MR‐guided treatment delivery. Diagnostic medical physicists have long‐supported MRI, but experience in design and delivery of continuing education suggests that some diagnostic physicists have a thorough knowledge of the fundamental physics of MRI, while many have not had the opportunity to study it in a structured setting. For nuclear medicine physicists, MRI now plays a key role as PET/MRI has become an established (if slow‐growing) hybrid imaging modality. The duties of all medical physicists are evolving to require deeper knowledge of MRI physics than what medical physics graduate education, based on CAMPEP minimum requirements, provides today.

The current CAMPEP standards for graduate education^2^ contain a list of MRI topics that are required for a core “fundamentals of medical imaging” course. While this provides all medical physics graduates with a preliminary exposure to MRI topics, anecdotal accounts of graduate students and residents suggest that many graduate programs address these topics in as brief an episode as a single lecture within a semester course. This treatment does not lend itself to a mathematically rigorous approach to the subject nor does it provide sufficient context and opportunity for students to connect the fundamental physical phenomena and concepts studied to the day‐to‐day tasks and problems they will face in supporting MRI in the clinical setting.

A recent review of course offerings on CAMPEP‐accredited graduate programs’ public websites revealed that about 25% of programs offer dedicated courses in MRI physics, and fewer (about 10%) offer advanced MRI courses.^3^ Among the programs offering dedicated courses, it is not clear how many include MRI coursework among the core program graduation requirements, although at least one program does require all graduate students in all tracks to take its dedicated introductory MRI physics course.

Ensuring that all medical physics graduate students receive robust, structured education in MRI physics would help to address important practical problems for the medical physics profession. At present, there is concern about the number and growth rate of diagnostic medical physics residency programs. One of the barriers to starting and accrediting residencies that build on pre‐existing on‐the‐job‐training structures (which in some instances have existed for decades) is lack of resources for administrative support to launch and maintain the program. Given that diagnostic residency programs (and would‐be programs) suffer from these constraints, it seems highly unlikely that they would have resources to remediate residents’ minimal didactic preparation in MRI physics, even as they attempt to provide formal, structured training to support MRI in clinical radiology. Some residents are fortunate enough to work closely with faculty with strong MRI expertise and gain deeper understanding, but this is by no means assured by the structure of residency programs or their governing CAMPEP standards.

Therapy physicists find themselves at an even greater disadvantage; since the widespread use of MRI in radiation therapy is a relatively recent development, there are relatively few seasoned MRI veterans working in therapeutic medical physics who are available to teach residents and early career medical physicists the MRI physics concepts and insights that were not taught in graduate school. Radiation oncology now requires greater and increasing support from physicists with MRI expertise, both to support its MRI needs directly and to provide MRI education to existing therapy physicists, who are learning MRI and adding it to their skill sets. Reportedly, radiation oncology departments are having difficulty finding medical physicists for these roles. Increasing the supply of physicists with education in MRI fundamentals at the graduate level should help to fulfill these needs over time.

With the growing use of MRI in radiation therapy, it is incumbent upon therapeutic physics residency programs to have the ability to teach MRI skills and applications to their trainees. Our current generation of practicing physicists is working to acquire these skills in practice and through continuing education, but leaving our current residents to do so after graduation contradicts the premise of all of our efforts to standardize clinical training in residency.

We should strongly encourage CAMPEP to take action to give robust MRI physics education a core role in every medical physics graduate degree. This is a principled, educationally sound proposal and constitutes good policy to address a number of practical needs facing the future sustainability and growth of the medical physics profession.

### 2.B Jing Cai, PhD

Recent advances in MRI for radiation therapy applications have triggered a burst of research developments and discussions on the related topics,^1^, ^4^, ^5^, ^6^, ^7^ sending a strong signal that MRI physics may require more in‐depth training in order to prepare medical physicists for the upcoming challenges. While I generally agree that MR physics education should be enhanced, I do not believe that it should be achieved by requiring CAMPEP graduate program standards to have dedicated courses in MR physics.

Current CAMPEP requirements detailed in AAPM Report No. 197 (Academic Program Recommendations for Graduate Degrees in Medical Physics) are considerably extensive in MRI education. In addition, the core‐and‐elective curriculum structure adapted by CAMPEP also provides a solution to flexibly address the need of more rigorous MRI education. In current CAMPEP requirements, MRI is part of the core subject in Fundamentals of Imaging in Medicine, taking up approximately one credit‐hour. It covers the essential yet broad aspects of MRI, including basic principles, hardware, basic image quality issues, basic pulse sequences, artifacts, safety, and quality control. Some CAMPEP programs also offer an MRI elective that covers advanced MRI topics, such as pulse sequence programming. Medical physics students graduated from a CAMPEP program are expected to have developed a solid understanding of the fundamentals of MRI.

Understanding that new knowledge and skills are required for implementing advanced MRI technology in radiotherapy, I would like to point out, however, that such new knowledge is not new in terms of MRI physics fundamentals. It is rather new applications of MRI physics in the new settings, such as response assessment using MRI as imaging biomarker. CAMPEP requirements should focus on teaching students MRI fundamentals so that they can apply the knowledge toward clinical practice, rather than teaching particular applications. That being said, one can include examples of radiation therapy applications in MRI teaching, which can simultaneously enhance the understandings of both MRI physics and radiation therapy applications. Furthermore, there are various and maybe more effective means to obtain advanced training in MRI, such as seminars, conference presentations, and online educational resources.

Among various MRI applications in radiotherapy, some are designed with narrow purpose, such as quality assurance programs for MRI‐Linac hybrid machine, etc. I believe that medical physicists with basic MRI training have the ability to learn necessary knowledge. Some applications are more challenging, such as pulse sequence optimization and functional MRI for treatment assessment, can be accomplished by consulting with experienced MR imaging physicists.^1^ In addition, the argument of requiring dedicated MRI course is largely based on the projection that MRI will become a mainstream imaging modality in the future. However, the clinical popularity of MRI‐guided radiation therapy is unclear and debatable. Many questions have been raised regarding the added value of MRI‐guided radiation therapy over current IGRT, for example, what are the relative merits of tumor tracking using cine MRI in different clinical situations vs simple breath‐hold?^4^ Ultimately, the additional financial burden of an add‐on MRI for radiotherapy has to be justified by proving whether these conceptual advantages can translate to any measurable increase in patient survival or reduction in treatment toxicities.^5^

It may be impractical to require dedicated MRI physics course, especially for MS‐level CAMPEP programs. CEMPEP accredited or not, a medical physics program first needs to meet university required academic terms and set the number of required credits for the degree comparable to those in other disciplines. With limited course hours, introducing new material and course requirements will inevitably pay the price of removing or shortening other required courses. Faculty recruitment and qualification for a dedicated MRI course are also an issue that may challenge many CAMPEP programs. Furthermore, very few medical physicists in our current work force, imaging or therapy, have much formal training in MRI physics and yet we are managing well, thanks to the current CAMPEP required training that “teaches a man to fish, instead of giving him one.” Therefore, the effort and cost of increasing requirements to provide dedicated MRI training might not be justified. For a large proportion of medical physicists with the MS degree who plan to pursue clinical practice in radiation therapy outside academic settings, they would likely not see these as relevant.

To summarize, although I agree that MRI training should be enhanced due to the increased role of MRI in radiation therapy, I do not believe that CAMPEP should require dedicated MRI physics course which I think is unnecessary, impractical, and ineffective.

## 3 REBUTTAL

### 3.A David W. Jordan, PhD

I strongly agree with my opposing colleague's assertion that graduate programs should focus their efforts on fundamental knowledge, providing students with the context to apply the knowledge to clinical practice. Indeed, the advanced training in MRI available from various sources often falls short in this regard; aimed at working clinical medical physicists, such seminars, webinars, and conference talks often focus on clinical applications, tools, and methods at the expense of fundamentals. There is simply no way for these programs to incorporate the fundamental rigor of a semester‐long graduate‐level didactic course in the time allotted, whether in an hour‐long webinar or a week‐long summer school.

The CAMPEP‐required courses in Fundamentals of Imaging in Medicine currently fall well short of the goal of providing all medical physics graduates with a solid foundation in MRI physics. The three‐credit‐hour course must cover a multitude of other topics, so it is unlikely that even a full third of the course is devoted to MRI. Furthermore, the course may or may not be taught by an MR expert in any given program. The elective‐course model may create opportunities increase MR knowledge among graduates, but this relies on elective courses being offered by the programs (currently a very small minority) as well as motivation and awareness on the part of students to enroll in them.

While imposing requirements to programs is never easy, “where there's a will, there's a way” and given sufficient priority, a topic can find its rightful place in the core requirements. Medical physics programs and CAMPEP requirements should (and do) adapt and evolve to the needs of the profession and the clinic, and MRI has taken on a new importance that continues to grow. Faculty recruitment will create demand and opportunity for qualified teachers.

It is true that very few current medical physicists have extensive formal MRI education and that many are managing their clinical MRI responsibilities well. However, for many years, it has also been true that relatively few medical physicists completed residency training, yet as a profession we have chosen to do a better job of making our clinical training structured, consistent, and clinically relevant by requiring residency for all board‐certified medical physicists. It is time to recognize the important and growing role of MRI in all areas supported by medical physics and ensure that same structure, consistency, and clinical relevance in the teaching of MRI physics for all medical physicists.

### 3.B Jing Cai, PhD

Dr. Jordan and I both agree that MRI is playing an increasingly important role in modern medical physics, especially in radiotherapy, and therefore, MRI physics education needs to be enhanced. What we disagree about is whether CAMPEP should require dedicated MRI physics course in order to achieve this goal. Dr. Jordan pointed out a problem in current MRI physics education is that “many graduate programs address these topics (of MRI physics) in as brief an episode as a single lecture within a semester course.” While agreeing on this observation, I think this problem reflects more about an issue of inadequate implementation of current CACMPEP requirement, rather than an issue of the need for extended MRI physics teaching. To address this problem, efforts should be made to ensure adequate coverage of MRI physics per CAMPEP requirement. For example, it could be helpful to reduce teaching variations by specifying explicitly the credit requirement for the MRI component in the core “fundamentals of medical imaging” course, such as 1 credit hour. But more importantly, efforts should be made to enhance MRI teaching quality by finding/recruiting the right faculty with strong MRI expertise. This, however, leads to my next point on the impracticalness of requiring a dedicated MRI course.

The problem of aforementioned variations in MRI teaching is likely a consequence of lack of necessary MRI expertise in teaching faculty. This issue, if not properly resolved, will only become more problematic if CAMPEP requires a dedicated MRI course. Dr. Jordan pointed out the challenges that some residency programs lack physicists with MRI expertise and pondered this as a compelling reason that MRI physics should be taught comprehensively during the graduate study, rather than leaving it to the residency study. I fully agree with my opponent on his observation of the MRI expertise disparity among different programs; it should be noted, however, that in many medical physics programs, it is the same group of people who are involved in the graduate teaching and residency teaching. Lack of MRI expertise in residency program most likely also implies lack of MRI expertise in graduate program teaching. Relocating the problem will not help solving it without tackling its root cause which is the disparity in MRI expertise in different medical physics programs.

The rational that CAMPEP should require dedicated MRI course because of the increased use of MRI in radiation therapy is somewhat falsely poised. As we know, CT is the most widely used image modality in radiation therapy, yet CAMPEP does not require a dedicated CT course in medical physics graduate program. History has shown us that medical physicists trained with CT fundamentals are able to learn and handle new, extended CT‐related technologies through proper on job training and various forms of continuing education such as conferences and seminars. It can be reasonably expected that our medical physicists can also achieve similar goals using similar approaches, provided they have solid education in MR fundamentals which are already covered by current CAMPEP requirement.
